# How the psychosocial context of clinical trials differs from usual care: A qualitative study of acupuncture patients

**DOI:** 10.1186/1471-2288-11-79

**Published:** 2011-05-25

**Authors:** Fiona Barlow, Clare Scott, Beverly Coghlan, Philippa Lee, Peter White, George T Lewith, Felicity L Bishop

**Affiliations:** 1 Health Experiences Research Group, Department of Primary Health Care, University of Oxford, Rosemary Rue Building, Old Road Campus, Headington, Oxford OX3 7LF. UK; 2 Complementary Medicine Research Unit, Aldermoor Health Centre, Aldermoor Close, Southampton SO16 5ST, UK; 3 School of Psychology, University of Surrey, Guildford, GU2 7XH. UK; 4 Building 45, University of Southampton, Highfield, Southampton, SO17 1BJ. UK

## Abstract

**Background:**

Qualitative studies of participants' experiences in randomised clinical trials (RCTs) suggest that the psychosocial context of treatment in RCTs may be quite different to the psychosocial context of treatment in usual practice. This is important, as the psychosocial context of treatment is known to influence patient outcomes in chronic illness. Few studies have directly compared the psychosocial context of treatment across RCTs and usual practice. In this study, we explored differences in psychosocial context between RCT and usual practice settings, using acupuncture as our model.

**Methods:**

We undertook a secondary analysis of existing qualitative interviews with 54 patients. 27 were drawn from a study of western and traditional acupuncture in usual practice (for a range of painful conditions). 27 were drawn from a qualitative study nested in an RCT of western acupuncture for osteoarthritis of the hip or knee. We used qualitative analysis software to facilitate an inductive thematic analysis in which we identified three main themes.

**Results:**

In usual practice, starting acupuncture was more likely to be embedded in an active and ongoing search for pain relief, whereas in the RCT starting acupuncture was opportunistic. Usual practice patients reported few uncertainties and these had minimal consequences for them. In the RCT, patients experienced considerable uncertainties about their treatment and its effectiveness, and were particularly concerned about whether they were receiving real (or fake) acupuncture. Patients stopped acupuncture only at the end of the fixed course of treatment in the RCT, which was similar to those receiving acupuncture in the public sector National Health Service (NHS). In comparison, private sector patients re-evaluated and re-negotiated treatments particularly when starting to use acupuncture.

**Conclusions:**

Differences in psychosocial context between RCTs and usual practice could reduce the impact of acupuncture in RCT settings and/or lead to under-reporting of benefit by patients in trials. New trial designs that ensure participants' experiences are similar to usual practice should minimise differences in psychosocial context and help attenuate these potentially confounding effects.

## Background

The psychosocial context of treatment is known to influence outcomes in chronic illness across both conventional and complementary medical settings. Systematic reviews have shown, for example, that physicians can enhance treatment outcomes by adopting a warm, friendly, reassuring manner [1]; and that patients' expectations contribute to clinical outcomes [2]. We use the term psychosocial context to refer to the constellation of cognitions and emotions that patients experience toward their condition and its treatment and mean this to incorporate (but not be restricted to) patients' hopes, fears, desires, expectations, and evaluations. The term also acknowledges the social nature of patients' experiences of treatment, and we conceptualise patients as embedded in both their own personal social networks and also those relationships involved in treatment (e.g. with practitioners). In this study we aimed to explore how the psychosocial context of one treatment (acupuncture) differs according to whether that treatment is received in usual care or in a research setting. We chose to focus on acupuncture as it is one of the most widely used complementary therapies [3] and psychosocial factors, whether specific to acupuncture or not, may make important contributions to its effectiveness [4,5]. In the UK, current political proposals to support the provision of complementary therapies on the National Health Service (NHS) [6] make it particularly timely to reconsider how to evaluate such therapies.

Randomised clinical trials (RCTs) remain the gold standard of medical research: they exhibit less bias than non-randomised trials [7] [although see also [8]]; they are considered essential in the evaluation of all new interventions [9]; and they feature strongly in hierarchies of evidence and recommendations for developing evidence-based guidelines [10-12]. Many design features of the explanatory RCT enhance internal validity, they strengthen the causal inferences that can be made from the data [13]. For example, random allocation of participants to intervention or control groups is designed to minimise all other differences between these two groups (on both known and unknown confounders) [14,15]. However, while enhancing internal validity some design features can simultaneously reduce external validity. For example, homogeneous samples can enhance the precision of estimates of treatment effects, but they also reduce the extent to which findings can be generalised to the wider population of interest, i.e. people who do not fit restrictive inclusion/exclusion criteria. Furthermore, design features might translate into important differences in psychosocial context between receiving a treatment as part of an RCT and receiving the same treatment as part of usual care. The procedures involved across these two settings can be very different, for example there are often more documents to read and sign in RCTs (e.g. lengthy patient information leaflets and consent forms) and for participants in an RCT there is the chance of being randomised to and receiving the "control" or "placebo" treatment. Even if the treatment itself is identical and delivered in the same way across these settings, or if the comparison treatment is not placebo but usual care (as in pragmatic trials [13]), the knowledge that one is participating in an RCT might fundamentally change one's experience of the target treatment. Such differences in psychosocial context would further limit the external validity of the RCT. This could be particularly crucial for those treatments, such as acupuncture, in which psychosocial factors probably make a large contribution to outcome.

Some insight into the psychosocial context of RCTs is provided by qualitative studies of patients' experiences. For example, Stone and colleagues show how patients' expectations in RCTs are multifaceted and modified over time, and are shaped both before and during trials by multiple factors [16]. Others have described how participants can struggle to make sense of researchers' technical concepts such as equipoise [17] and randomisation [17-19]. Researchers have also explored the experiences of patients and practitioners in acupuncture RCTs [20-23]. Some aspects of psychosocial context might, by definition, be unique to RCT settings (for example concerns about placebos and placebo responding [16]). Other aspects, such as holistic consultation processes, might be attenuated in the RCT setting [21]. However, qualitative studies that focus solely on RCT participants can only tell us about the psychosocial context of RCTs, they cannot tell us about the extent to which the psychosocial context of RCTs differs from the psychosocial context of usual care. There are few direct comparisons between patients' experiences in RCT and usual care settings that could further elucidate these issues. We could locate one such study in acupuncture, which focused on experiences of holism rather than psychosocial factors more broadly [24]. Therefore, we conducted this study to compare directly patients' experiences of acupuncture within an RCT and everyday clinical practice. The aim was, in the context of acupuncture, to identify any differences in psychosocial context between an RCT and usual care setting. The related objectives were to explore the nature of any such differences and to examine implications for trial methodology.

## Method

### Design

This is a secondary analysis of interviews with patients about their experiences of acupuncture. Interviews were drawn from a stand-alone qualitative study of western and traditional acupuncture for pain in usual care [25] and a qualitative study of western acupuncture for osteoarthritis pain that was embedded within an RCT [20]. All participants gave written informed consent before being interviewed and consented to the publication of anonymous quotations.

### Procedure

#### RCT Study

Face-to-face open-ended narrative interviews were conducted by CS with 27 participants in a large RCT of western acupuncture for osteoarthritis pain. This factorial trial investigated specific and non-specific effects and randomised participants to one of three interventions: real needles, Streiberger (placebo) needles and mock electrical stimulation (a non-needle placebo); one of two consultation types: empathic and non-empathic; and one of three practitioners. Participants were recruited from hip or knee- joint replacement waiting lists and some had previous experience of acupuncture before joining the trial. All received eight, 30 minute treatment sessions over a 4 week period. A purposive sample of participants, including patients from all intervention arms, was invited to take part in an interview four to eight weeks after completion of the trial treatments. The opening question asked interviewees to talk about their experiences of taking part in the trial. There was no topic guide, but follow-up probes were used to encourage elaboration. The interviews typically lasted approximately 60 minutes (range 30-120 minutes) and were audio-taped, transcribed, and supplemented with field-notes. Summaries were sent to the interviewees. This study received approval from Southampton & South West Hampshire and the Salisbury and South Wiltshire Research Ethics Committees (approval number 170/03/t).

#### Usual Practice Study

Face-to-face open-ended semi-structured interviews were carried out by BC and PL with 27 people receiving acupuncture for pain at clinics in the south of England. To be eligible, people had to have had recent experience (in the past 2 years) of using traditional or western acupuncture for pain in either the NHS or private practice (or both). (In the UK, acupuncture is predominantly used in the private sector but is also accessible in some parts of the NHS.) Those who had had previous earlier experiences of acupuncture *in addition to *their recent experiences were also eligible. A concerted effort was made to interview men and women of a range of ages and who reported a variety of painful conditions, people who reported positive and negative experiences of acupuncture and those who had experienced acupuncture within the NHS and within private practice (i.e. a maximum variation sample). Interviews began with a broad, open question designed to elicit participants' narratives ("I'm really interested in finding out all about your experiences of having acupuncture; please can you tell me all about it?"). This was followed up if necessary with prompts to talk about feelings before having acupuncture, experiences of treatments and consultations, the impact of acupuncture, and the provision of acupuncture (e.g. on the NHS or in private practice). The interviews typically lasted approximately 60 minutes (range 35-105 minutes) and were audio-taped, transcribed, and supplemented with field-notes. Summaries were sent to the interviewees. This study was approved by the Southampton and South West Hampshire Research Ethics Committee (B) (reference number 07/HO504/196).

### Participants

Participants' characteristics are shown in Table 1. More women than men were interviewed in both settings. The usual practice study included participants with a range of painful conditions, not just osteoarthritis of the hip or knee. We have included trial participants who received placebo acupuncture as well as those who received real acupuncture because these participants did not differ in relation to the main themes reported below.

**Table 1:** Participants' Characteristics

Characteristic	Trial (n = 27)	Usual practice (n = 27)
Gender: Female	18 (67%)	20 (74%)
Paid out of pocket	0	14 (52%*)
Received real acupuncture	8 (30%)	27 (100%)
Primary Condition(s)	Osteoarthritis hip or knee	Chronic pain including pain in foot, knee, back, neck, shoulder.

* A further 7 participants had some experience of paying out of pocket for acupuncture but had also received acupuncture on the NHS

### Analytic Methods

This secondary analysis was led by a researcher who had not been involved in either of the original studies (FB). All 54 interviews were analysed using Atlas Ti software and commonalities and differences between the RCT and usual practice patients' experiences were explored. An inductive thematic analysis was undertaken following Braun and Clarke [26] and incorporating established techniques for coding drawn from Grounded Theory [27,28]. The researcher familiarised herself with the data by repeatedly reading the interview transcripts and field notes. The researcher then annotated the transcripts with inductive codes (developed from the data) that summarised and conceptualised participants' experiences. Each unit of speech (e.g. a phrase or sentence) that described an aspect of a participant's experience of acupuncture was annotated in this way. This detailed analysis was carried out on all transcripts individually. As coding proceeded, a process of constant comparison was undertaken in which the researcher compared different codes with each other, compared different phrases that had been annotated with the same codes, and compared the codes that were applied to RCT interviews to those that were applied to the usual practice interviews. Through this process of comparison, similar codes were grouped together to form categories and higher level (more abstract) themes were identified that described key differences in the psychosocial context of acupuncture between the RCT and usual practice. Initial coding and emergent themes were discussed in detail with a second researcher (FLB) who also undertook a final check of the analysis against all original transcripts to ensure key points were covered and patients' experiences were summarised accurately.

While our primary concern was to explore differences in the psychosocial context for patients when they received acupuncture as part of a trial or usual practice, it is important to note that, according to the interviewees' descriptions, the procedures for administering acupuncture within the trial, NHS and private practice were very similar (although in private practice the practice nurse was often responsible for the removal of the acupuncture needles whereas in the trial and the NHS the acupuncturist removed the needles). Three main themes were identified that describe the key differences in psychosocial context between the RCT and usual practice. These themes are described below with illustrative quotes selected for typicality and vividness. Pseudonyms are used for each participant to give a more personal portrayal of the quotes while ensuring anonymity.

## Results

### Starting Acupuncture: Actively Seeking Treatment

Participants in the RCT and usual practice took different pathways to starting acupuncture. Many of the usual practice patients who were paying for their treatment reported prior experience of acupuncture and returned to it because it had been successful for them in the past. For some private sector patients and most NHS patients, acupuncture was a new experience which they had been given on the recommendation of their healthcare provider (typically NHS patients who were seeing physiotherapists or pain clinic doctors) or a family member or friend (typically private patients). Dan's story was typical of patients having acupuncture in usual practice in the NHS.

"They all work at the pain clinic, so I mean, you go in there, you'll see one or either of them and they might refer you to somebody else and that's how I got this [acupuncture]. First I went to see the consultant and then they put us in touch with the physiotherapist and then the physiotherapist put us in touch with the acupuncturist." (Dan, Usual Practice, NHS)

Usual practice patients were seeking and expected varying degrees of pain relief (from cure to some relief) and hoped that they would be able to decrease the amount of analgesics they were taking. Those in the private sector also reported wanting help to cope with the pain and seeing acupuncture as a means to enhance their natural healing. For some patients in usual practice, such as Pat, acupuncture was one in a long series of attempts at pain relief, and they tried acupuncture "out of sheer desperation" and "as a last resort".

"[I went for acupuncture] really in desperation at being unable to get rid of some focal, not disabling, but really severely debilitating aches in both elbows that I've been having for, probably getting on for a year, and I'd used various remedies including quite strong pain killers which had an effect, with limited duration, um, topical tiger balm and topical NSAIDs without any success" (Pat, Usual Care, Private).

In comparison to the usual care patients, those who took part in the RCT were all recruited from the waiting list for the surgical replacement of hip or knee joints (14 knee replacements/13 new hip joints). Some had prior experience of acupuncture but a majority did not and so in this way they resembled the patients having acupuncture as usual care in the NHS. All were responding to a letter of invitation they had received from the hospital- based research team, and so received rather than sought out the opportunity to start acupuncture. Their reasons for taking part in the study appear more diverse than the usual practice patients. Although, like patients in the usual practice group, trial patients hoped acupuncture would alleviate their pain and possibly decrease their use of analgesics, several patients also felt it was an opportunity to try acupuncture for free while awaiting surgery and a couple of patients saw acupuncture as a possible means of delaying or even avoiding surgery. Typically, trial participants felt they had nothing to lose by trying acupuncture, as they knew they would (or could) receive surgery in due course. Indeed, the trial offered participants the chance to access a treatment that might help them to cope with their pain while remaining on the surgical waiting list. Some patients, such as Stella, reported altruistic reasons for taking part in the trial (e.g. to help the researchers and future patients) and emphasised starting the trial rather than starting acupuncture per se.

"I decided to take part because I was experiencing so much discomfort, and I knew of other ladies who were waiting for it [joint replacement surgery] who were experiencing even worse pain than I was. And I thought if I can go and get involved in this research programme that may bring it into the National Health to help these other ladies. Or the ladies of the future that, you know, would be waiting for the operation. When you're in pain and they decide that you need this operation and they tell you its six months you think "Oh my God I've got to put up with this for another six months". That was the real...I thought I was going to help get acupuncture into the hospital so that other people could be relieved of the pain. That's the reason I did it." (Stella, RCT, receiving non-needle placebo acupuncture)

### During Treatment: Uncertainties and Anxieties

Patients in the RCT expressed uncertainties over the course of treatment to a greater extent than did patients in usual practice (see Table 2). Many patients sought to confirm the value of acupuncture either at the start or during their time on the trial. As with the usual practice patients, many RCT patients received information from their healthcare professional or significant others. Unlike the usual practice patients, RCT patients were also given a detailed patient information sheet in which they were made aware of the possibility of receiving placebo treatments. All hoped to receive benefit from acupuncture, but were aware that they might be given placebo.

**Table 2:** Uncertainties during Treatment

Setting	Uncertainties
Usual Practice	"It wasn't instant relief, no, it was a gradual improvement. Like, I think, all alternative medicine, it's you know, it's a big fly reel, you start you don't notice any change but gradually you do start noticing a change and then it can keep you going a long time." (Paul, Private)
	"I don't know how it's suppose to work or what it's doing all I know is that I think it is good for me for whatever reason, I don't know if acupuncture works for everybody or there are certain people it works on or I don't know but certainly for me um...it works." (Jane, Private)
	"I suppose I'm not convinced how much [improvement] is actually related to the acupuncture and how much is just chance, because ... it does go up and down in terms of how bad it is and, you know, it's only 12 days, since I had the first lot, therefore it could just be chance that this is coming to, like, a good bit." (Joanne, Private)

Trial	"You never know whether you are getting the right treatment, or if it's a dud you're taking, do you?" (Sidney, receiving placebo needles)
	"I think I would have felt a fraud had it been placebo and there had been this change which there is. I think I'd have thought then there must be a process in my head which...and I didn't like that because again it doesn't fit with my idea of exactness and those sort of things." (Norman, receiving real acupuncture)
	"The only thing that you do think about is - you're having your treatment and you're not sure if you are having something that is supposed to do something or whether you're just having something that's just sticking needles in you..... And you're always a bit hesitant to say "well I think its doing me good" because you've got that feeling that perhaps what you're having done isn't...you know." (Martin, receiving real acupuncture)

Whilst both usual care and trial groups monitored the effects of their treatments and assessed whether treatments alleviated their pain, the trial group also monitored their treatment process to confirm or deny the veracity of their treatments. This is a crucial difference between trial and usual care patients. Usual practice patients were confident that they were receiving the acupuncture they had elected to receive, whilst RCT patients knew they might be receiving a placebo. The uncertainty regarding the treatment they were receiving within the trial led patients to express a fear of being duped or looking a fool, if they declared improvements after receiving placebo. These participants' experiences were imbued with a lack of trust in their own pain sensations, and their reports of improvements were often expressed with the caveat that theirs might be a placebo response.

There was less evidence of uncertainty experienced by patients receiving acupuncture in usual practice. Any uncertainties typically featured in discussions of initial acupuncture treatments, or short courses of acupuncture, when participants were as yet unsure as to whether they were receiving any benefit. Occasionally participants expressed uncertainty about other aspects of acupuncture, such as its mechanisms of action. However, the consequences of these uncertainties were far less complex and extensive than those experienced by RCT participants. Typically, when usual practice participants had experienced uncertainty about the impact of acupuncture they decided to have more treatments to help them find out whether they could benefit from it. Uncertainty about its mechanisms of action had no apparent consequences, as participants cared more about whether acupuncture worked than how it worked.

### Continuing and Stopping Acupuncture: Flexibility and Rigidity in Committing to a Course of Treatment

Usual care patients in the private sector decided for themselves (or in collaboration with their acupuncturist) whether to continue or stop receiving acupuncture. They typically based these decisions on whether they felt they were benefitting from acupuncture and, as Dannie illustrates, had the flexibility to experiment with the timing of their treatments.

"We tried to go on a sort of monthly basis and then extend it and see how long I could last, um, which was quite interesting to see how long I could last without getting my symptoms back, the pains back, and um sometimes it could be six weeks, even two months and then other times, if I was under a lot of stress or doing a lot of work, um, I'd have to go more often but to begin with I think it was um quite relevant that to begin with I had to go very frequently and quite quickly I was able to leave it a whole month and that seemed to indicate to me a great success." (Dannie, Usual Care, Private)

Whilst it is expected that usual practice patients in the private sector who were not benefiting from acupuncture would stop treatment, trial patients who did not perceive they were gaining any benefit from their acupuncture continued to attend for their allotted number of treatments. Although some guessed they were receiving placebo, they gave altruistic reasons for completing the trial.

"I was not convinced at any time that this was having any benefit whether real or not, whatever you were attempting to do, but err, I was prepared to give it a go because it might benefit somebody." (David, RCT, receiving non-needle placebo acupuncture)

In terms of continuing with a fixed course of treatment, trial patients were similar to those receiving acupuncture in the NHS who typically signed-up for a course of treatments. They did not revisit this decision until the end of the course, at which point it was often not possible to access more treatments on the NHS. Those patients who felt they had benefitted and wanted to continue acupuncture then sought to continue treatment in the private sector, except when the financial cost was an insurmountable barrier, as Joanne described: "I also can't afford to run over my limit. So money is a factor."

## Discussion

We identified differences in the personal motivation and broader psychosocial context of acupuncture when it was experienced in an RCT compared to usual care. RCT participants and usual care patients hoped acupuncture would provide personal pain relief, but RCT participants also volunteered to help 'someone else' and to assist the researchers. Patients in usual care did not report any such additional reasons for starting acupuncture. Before treatment, trial participants had received detailed information about the trial from the investigators - including the possibility that they could receive placebo - and most had discussed the invitation to take part with friends and relatives. Usual care patients had received recommendations and/or testimonials in person from their existing health care professional or a friend/relative. For those trying acupuncture in usual care (and especially in private practice), the decision to have acupuncture was more embedded in an ongoing personal quest for pain relief. This is consistent with existing literature about the importance of personal recommendations and social networks in seeking acupuncture and other forms of complementary medicine [29-32]. For those trying acupuncture in the RCT the decision was more opportunistic and, while often consistent with underlying beliefs, was not central to an ongoing quest for pain relief as these patients were reassured by the knowledge that they would soon be undergoing joint replacement surgery. This particular finding is linked to the context of this particular trial - which recruited from surgery waiting lists - but the general pattern does suggest that RCT participants may be less engaged with the whole process of acupuncture treatment (at least initially) than patients who actively seek it out in the community.

During treatment, trial participants were concerned and sometimes anxious about the possibility that they were receiving placebo rather than real acupuncture. Usual care participants experienced no such anxieties. Both groups of participants monitored the impact that acupuncture was having on them. Those receiving acupuncture in usual care monitored its impact in order to inform decisions to continue or to stop acupuncture; previous studies of complementary therapies in usual care report similar findings [33-35]. Those receiving treatment in the RCT monitored its impact to help them figure out whether they were receiving real or placebo acupuncture; previous studies of RCTs also describe patients' concerns about receiving placebo [22]. Patients receiving acupuncture in usual care were confident in expressing any perceived benefits and based their ongoing use of acupuncture on their perceptions of benefit. However, those receiving acupuncture in the RCT were afraid of appearing foolish, and were anxious about reporting benefit when they may actually have received placebo rather than real acupuncture. This difference could lead to considerable under-reporting of benefit by patients in trials compared to usual practice. In addition, the extra concerns that the RCT patients had about placebos would be likely to distract them from the treatment process and might mean that they were less engaged in this process than patients in usual care. As has been discussed in the literature, engagement with the process of traditional acupuncture might be particularly important given that acupuncturists strive to provide holistic care for example incorporating self-management advice related to diet and exercise [36,37].

Our final key finding related to continuing and stopping acupuncture. In usual care, participants stopped having acupuncture if they perceived no benefit and continued to have acupuncture if they perceived benefit, if they hoped to obtain benefit, or if they relapsed on stopping acupuncture. In the RCT, participants continued having acupuncture until the end of the trial. Indeed, in the trial as a whole only 4.9% of participants dropped out before the end of treatment. While there is very little data about adherence to acupuncture regimens in usual care, our findings suggest that RCT participants may be more likely to continue a course of acupuncture treatment even in the absence of benefit than patients receiving acupuncture in usual care (particularly those paying for treatment). As a consequence of such behaviour, RCTs might underestimate both effectiveness and cost-effectiveness of acupuncture compared to usual practice. This hypothesis requires testing in future work.

There are some limitations to our work. In this qualitative study we chose to explore differences in patients' experiences between RCT and usual care in the very specific context of acupuncture. Our findings are grounded in this context but we hope they offer an illustrative and informative example of the ways in which patients' psychosocial contexts can differ across RCT and usual care. Consequently the findings may have relevance for similar situations such as trials of other physical therapies for painful conditions. There were a few methodological differences between the two original qualitative studies: interviews were carried out by an acupuncturist-PhD student in the RCT study and by one undergraduate medical student and one postgraduate psychology student in the usual care study. Unlike in the RCT study, the interviews in the usual care study did not explicitly take a narrative approach. We maintain that our secondary comparative analysis is appropriate despite these differences because the interviews were similar in scope, all used open-ended questions, and were similar in duration and depth: interviewees described similar aspects of acupuncture experiences in similar detail in each study. Our trial participants had taken part in a complex factorial placebo-controlled trial of acupuncture for osteoarthritis pain, which incorporated an empathy manipulation as well as two placebos. It is possible that the complexity of this particular trial created more uncertainty for participants than might be experienced in other trials. Finally, the acupuncture treatments that participants experienced in the RCT and in usual care were not themselves identical. For example, some of the patients in usual care had experienced traditional acupuncture whereas all of the patients in the RCT received Western acupuncture. It is therefore possible that the differences we identified were due to differences in the acupuncture treatments/consultations, rather than the differences in setting. However, given the consistent nature of the differences we identified we feel this latter explanation is unlikely.

Our work has implications for medical research methodology and for the interpretation of published trials. Our analysis provides an initial illustration of how RCTs can lack ecological validity because of the different psychosocial context that is created for and by patients. The complexity of inter-relationships between psychosocial context and outcomes make it difficult to be confident about the clinical implications of these differences. However, our findings are consistent with the suggestion that the psychosocial context of usual care might be more likely to enhance acupuncture compared to the psychosocial context of the RCT [5].

Pragmatic trials might avoid some of the contextual idiosyncrasies that we identified (namely placebo anxiety). However, participants in pragmatic trials might still be concerned to identify their treatment allocation, and other contextual differences probably remain (e.g. around differing levels of engagement with the target intervention). A recent innovation, the cohort pragmatic trial [38], involves recruiting a large cohort to an observational study and then randomly selecting a sample to receive the target intervention. This design thus enhances the similarities between the clinical trial context and real world practice and so ought to attenuate differences in psychosocial context between trial and usual practice. Future research could address the extent to which specific trial procedures (e.g. patient information sheets, consent interviews) contribute to the psychosocial context of trials and thus offer opportunities to further attenuate differences between trials and usual practice.

## Conclusions

Our work in acupuncture suggests that, compared to patients receiving treatment in usual practice, patients who receive treatment in an RCT may be less engaged with it, are motivated to participate in a trial as well as to receive treatment, appear to experience more concerns and doubts, and may be more likely to finish a course of treatment despite lack of perceived benefit. Such differences could reduce the impact of treatment in RCT settings and/or lead to under-reporting of benefit by patients in trials. New trial designs that make trial participants' experiences more similar to usual practice should minimise differences in psychosocial context and help attenuate these effects.

## List of Abbreviations

RCT: Randomised clinical trial; NHS: National Health Service

## Competing interests

The authors declare that they have no competing interests.

## Authors' contributions

FLB conceived of and designed this secondary study, participated in the analysis, and drafted the manuscript. FB led this secondary analysis. BC and PL carried out the interviews with the usual practice participants, under the supervision of FLB and GTL. CS carried out the interviews with the RCT participants, under the supervision of PW and GTL. PW and GTL contributed to the design and analysis. All authors were involved in revising the manuscript and read and approved the final manuscript.

## Pre-publication history

The pre-publication history for this paper can be accessed here:

http://www.biomedcentral.com/1471-2288/11/79/prepub
